# Caspase-8 controls the gut response to microbial challenges by Tnf-α-dependent and independent pathways

**DOI:** 10.1136/gutjnl-2014-307226

**Published:** 2014-06-24

**Authors:** Claudia Günther, Barbara Buchen, Gui-Wei He, Mathias Hornef, Natalia Torow, Helmut Neumann, Nadine Wittkopf, Eva Martini, Marijana Basic, André Bleich, Alastair J M Watson, Markus F Neurath, Christoph Becker

**Affiliations:** 1 Medical Clinic 1, Friedrich Alexander University, Erlangen, Germany; 2 Institute for Medical Microbiology and Hospital Epidemiology, Hannover Medical School, Hannover, Germany; 3 Institute for Laboratory Animal Science and Central Animal Facility, Hannover Medical School, Hannover, Germany; 4 Norwich Medical School, University of East Anglia, Norwich, UK

**Keywords:** Apoptosis, TNF, Cell Biology, Cell Death, Intestinal Epithelium

## Abstract

**Objectives:**

Intestinal epithelial cells (IEC) express toll-like receptors (TLR) that facilitate microbial recognition. Stimulation of TLR ligands induces a transient increase in epithelial cell shedding, a mechanism that serves the antibacterial and antiviral host defence of the epithelium and promotes elimination of intracellular pathogens. Although activation of the extrinsic apoptosis pathway has been described during inflammatory shedding, its functional involvement is currently unclear.

**Design:**

We investigated the functional involvement of caspase-8 signalling in microbial-induced intestinal cell shedding by injecting Lipopolysaccharide (LPS) to mimic bacterial pathogens and poly(I:C) as a probe for RNA viruses in vivo.

**Results:**

TLR stimulation of IEC was associated with a rapid activation of caspase-8 and increased epithelial cell shedding. In mice with an epithelial cell-specific deletion of caspase-8 TLR stimulation caused Rip3-dependent epithelial necroptosis instead of apoptosis. Mortality and tissue damage were more severe in mice in which IECs died by necroptosis than apoptosis. Inhibition of receptor-interacting protein (Rip) kinases rescued the epithelium from TLR-induced gut damage. TLR3-induced necroptosis was directly mediated via TRIF-dependent pathways, independent of Tnf-α and type III interferons, whereas TLR4-induced tissue damage was critically dependent on Tnf-α.

**Conclusions:**

Together, our data demonstrate an essential role for caspase-8 in maintaining the gut barrier in response to mucosal pathogens by permitting inflammatory shedding and preventing necroptosis of infected cells. These data suggest that therapeutic strategies targeting the cell death machinery represent a promising new option for the treatment of inflammatory and infective enteropathies.

**Significance of this study**

**What is already known on this subject?**

- Loss of intestinal barrier function plays an important role in the pathogenesis of IBD.
- Tnf-α and toll-like receptor (TLR) stimulation can induce a transient increase in epithelial cell shedding with loss of epithelial barrier function.
- Escape from apoptotic cell death due to caspase-8 deficiency results in necroptosis of Paneth cells at the crypt base. The role of necroptosis in cell shedding from the villus is unknown.

**What are the new findings?**

- Caspase-8 controls cell shedding and cell death on the villus induced by TLR ligands in vivo.
- When caspase-8-dependent apoptosis is disabled, cell death and cell shedding switch from apoptosis to Rip3-dependent necroptosis causing villous destruction which is more severe than from apoptosis.
- Blockade of necroptosis prevents mortality and cell shedding following TLR stimulation, and preserves mucosal integrity.
- Tnf-α produced by non-epithelial cells is responsible for TLR4-mediated epithelial necroptosis.
- TLR3-triggered necroptosis is directly mediated via TRIF-dependent pathways, and independent of Tnf-α and type III interferons.

**How might it impact on clinical practice in the foreseeable future?**

- This study expands the current knowledge of regulation of apoptotic and necroptotic pathways controlling cell shedding from intestinal villi. Our findings show that therapies that inhibit apoptosis must be accompanied by manoeuvres that also inhibit necroptosis. This has important implications for the design of treatment of inflammatory and infectious intestinal diseases and, thus, for clinical practice.

## Introduction

The gut represents the largest surface of the human body to the external environment, which is characterised by food antigens and the mucosal microbiota, including bacteria, viruses and parasites. Intestinal epithelial cells (IEC) constitute an important barrier in controlling the access of luminal antigens into the body. Owing to these essential functions, it is quite evident, that the intestinal epithelium plays a crucial role for intestinal homeostasis as well as for systemic health. Dysfunction of the intestinal epithelium, as indicated by increased permeability or deregulated epithelial defence functions, is believed to result in the excessive translocation of the commensal microbiota into the bowel wall and subsequent development of gastrointestinal diseases, including IBD.^1–3^ In this context, several studies have demonstrated increased apoptotic epithelial cell death in patients with IBD.^4–7^ Recent studies have shown that a second form of regulated cell death, denoted as necroptosis, occurs in human IBD. Necroptosis is a Rip3-dependent cell death pathway that is particularly prominent in Paneth cells at the crypt base which leads to loss of defensin secretion and IBD.

Intestinal epithelial homeostasis is maintained by a strict equilibrium between cell proliferation in the crypt and cell shedding from the villus tip.^8^ Stimulation with tumor necrosis factor (TNF) or LPS increased apoptosis and consequent cell shedding and is associated with barrier loss.^9–11^ Excessive cell shedding and barrier loss in IBD patients in remission predicts relapse.^12^ However, the role of toll-like receptors (TLRs) in the regulation of cell shedding is poorly understood and the role of necroptosis in cell shedding and barrier loss has not been reported.

Here, we demonstrate that activation of TLR3 and TLR4 leads to a rapid and transient activation of caspase-8 and caspase-3 as well as increased cell shedding. Surprisingly, deletion of caspase-8 from the intestinal epithelium did not protect the animals from TLR-induced inflammatory cell loss, but instead, led to severe tissue destruction, excessive cell death and mortality due to Rip3-dependent necroptosis. Our analysis further revealed that TLR3-triggered necroptosis was directly mediated via TIR-domain-containing adapter-inducing interferon-β (TRIF)-dependent pathways but independent from Tnf-α and type III interferons, whereas LPS-induced necroptosis was strictly Tnf-α dependent. Therefore, from our data, we propose a novel model of villus cell shedding in response to a range of inflammatory stimuli in which the activation of caspase-8 is essential during the first line of defence, by maintaining the gut barrier in response to mucosal pathogens through induction of inflammatory shedding and inhibition of necroptosis in infected cells.

## Materials and methods

### Mice

Rip3^−/−^mice, Tnf-R1^−/−^ mice, TRIF^−/−^ mice, IL-28Rα^−/−^ mice, VillinCre mice and mice carrying loxP-flanked *caspase-8* alleles were described earlier.^13–16^ Conditional knockout mice (Casp8^ΔIEC^ mice) were generated by breeding floxed caspase-8 mice to VillinCre mice as described earlier.^13^ Mice were routinely screened for pathogens according to FELASA guidelines. Animal protocols were approved by the Institutional Animal Care and Use Committee of the University of Erlangen.

### Experimental models of systemic inflammatory response syndrome

Mice were injected intraperitoneal with LPS (2.7 mg/kg body weight, Sigma-Aldrich) or poly(I:C) (30 mg/kg or 35 mg/kg for experiments with TRIF^−/−^ mice, InvivoGen). Viability was monitored by visual examination and by measuring body temperature and weight loss.

### Histology and immunohistochemistry

Histopathological analysis was performed on formalin-fixed paraffin-embedded tissue using the Tyramide Signal Amplification (TSA) Cy3 system, as recommended by the manufacturer (PerkinElmer), and the following antibodies were used: the primary antibodies anti-Myeloperoxidase (MPO) (Abcam), anti-Tnf-α (Cell Signaling), anti-activated caspase-3 (R&D Systems) and anti-activated caspase-8 (Cell Signaling) and anti-CD324 (E-cadherin) Alexa Fluor 488 (eBioscience), and a biotinylated secondary anti-rabbit antibody (Dianova). Cell death was analysed using the in situ cell death detection kit (Roche) for TUNEL.

### Immunoblotting

Proteins were separated using a MiniProtean Precast gel (4–15% polyacrylamide; BioRad) and transferred to a nitrocellulose membrane (Whatman). Membranes were probed with the following primary antibodies: TLR3 (Santa Cruz), Rip3 (Enzo), cleaved caspase-3, cleaved caspase-8 XP (Cell Signaling) and actin (Abcam). HRP-linked anti-rabbit (Cell Signaling) was used as a secondary antibody.

### Gene expression

Total RNA was extracted from the gut tissue, or organoids, using the RNA isolation kit (Nucleo Spin RNA II, Macherey Nagel). cDNA was synthesised by reverse transcription (SCRIPT cDNA Synthesis Kit, Jena Bioscience) and analysed by real-time PCR with SYBRGreen (Roche) reagent and QuantiTect Primer assays (Qiagen). Experiments were normalised to levels of the housekeeping gene hypoxanthine guanine phosphoribosyl transferase (*HPRT*).

### Crypt isolation and organoid culture

For organoid culture, crypts were isolated from the small intestine of mice and cultured for a minimum of 7 days as previously described by Sato and Clevers.^17^ Organoid growth was monitored by light microscopy and Propidium Iodide (PI) staining (2 µM, BD Biosciences). Organoids were treated with LPS (450 ng/mL, Sigma-Aldrich), poly(I:C) (600 ng/mL, InvivoGen), necrostatin-1 (30 µM, Enzo) or zVAD-fmk (120 µM, Bachem).

### Statistical analysis

Statistical analysis was performed using the two-tailed Student t test. *p≤0.05, **p≤0.01, ***p≤0.001.

## Results

### Epithelial TLR stimulation promotes caspase activation in the absence of cell death

**Figure 1:**
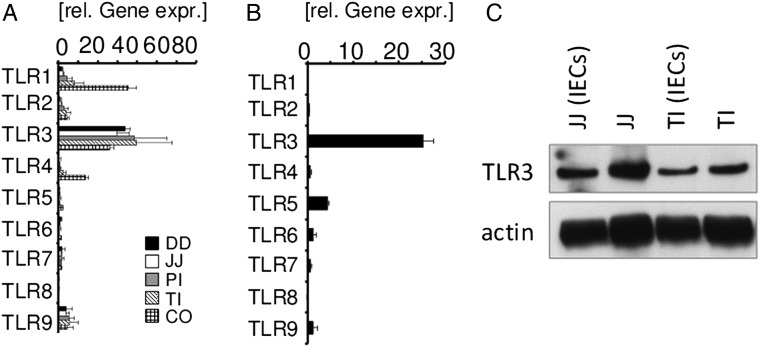
Toll-like receptors (TLR) expression in the intestine. (A) Quantitative expression levels of *TLR1-9* mRNA in bowel segments of healthy wild-type mice (n=3). DD, duodenum, JJ, Jejunum, PI, proximal ileum, TI, terminal ileum, CO, colon (+SD, relative to hypoxanthine guanine phosphoribosyl transferase, *HPRT*). (B) Quantitative expression level of *TLR* mRNA in organoids derived from the small intestine of healthy control mice (n=6, +SD, relative to *HPRT*). (C) Immunoblot analysis of TLR3 in the small intestine of a healthy control mouse: whole tissue of JJ and TI and isolated Intestinal epithelial cells (IEC) from both parts (JJ, IECs) and (TI, IECs). Actin staining was added as a loading control.

IECs express TLRs, endowing them with the capacity to directly respond to microbial challenges.^18^ ^19^ As TLR signalling in IEC has been found to regulate epithelial cell shedding and has been implicated in the pathogenesis of IBD, we investigated the molecular mechanisms of such inflammatory cell elimination. Since microbial colonisation is heterogeneous along the gastrointestinal tract, we determined in an initial series of studies the expression level of *TLR1-9* in different parts of the intestine (figure 1A). We observed a marked expression of *TLR3* mRNA in all bowel segments, whereas *TLR1*, *TLR2*, *TLR4* and *TLR5* mRNA expression was mainly found in the distal segments with the highest concentration in the colon. The analysis of *TLR* expression in epithelial stem cell-derived organoids obtained from wild-type mice confirmed the epithelial nature of intestinal *TLR3* expression in the intestine (figure 1B). Additionally, immunoblot analysis of small intestinal samples from healthy wild-type mice supported the expression of TLR3 in IECs (figure 1C). To evaluate the capacity of microbiota-derived TLR ligands to induce cell shedding and mucosal damage, a sublethal dose of LPS, or Polyinosinic polycytidylic acid (poly(I:C)), an immunostimulant that simulates viral infection and interacts with TLR3 was injected intraperitoneally into wild-type mice. Strikingly, administration of either LPS or poly(I:C) resulted in rapid epithelial cell shedding (see online supplementary figure S1A). Of note, the most pronounced TLR ligand-induced tissue damage was observed in the small intestine as demonstrated by quantitative RT-PCR for the IEC marker villin (see online supplementary figure S1B,C). TLR-induced cell shedding was associated with a rapid activation of caspase-8 and caspase-3 in small intestinal tissue sections suggesting an involvement of the latter proteases in this process (see online supplementary figure S1A). Thus, activation of the extrinsic apoptosis pathway is observed during TLR ligand-induced epithelial cell shedding, and may be involved in tissue damage and inflammation observed upon microbial challenge of the epithelial barrier.

### Epithelial caspase-8 minimises mortality after TLR stimulation

**Figure 2:**
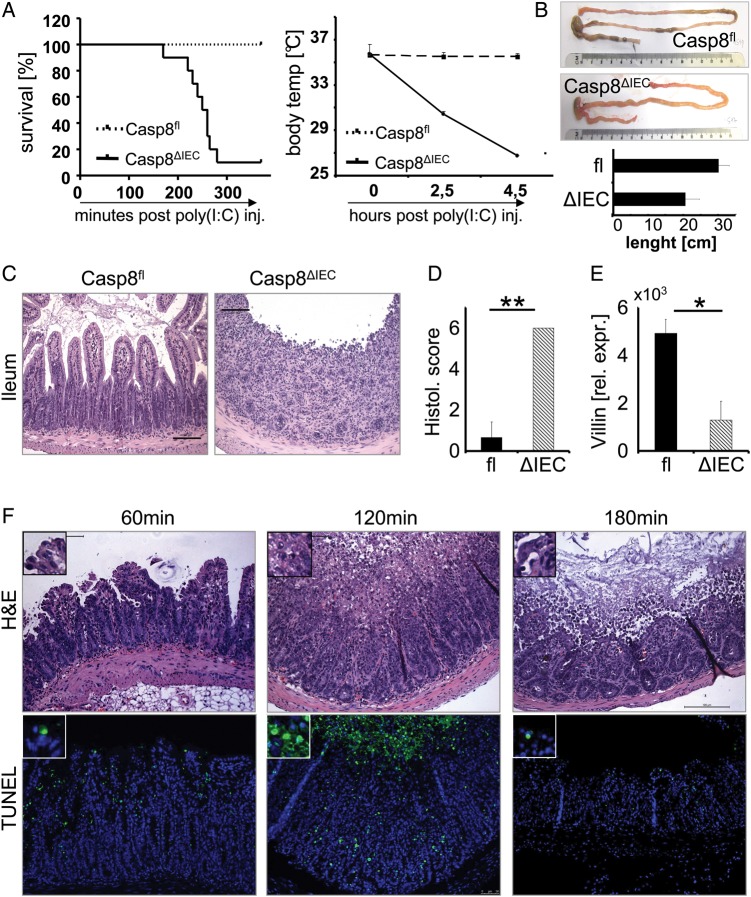
Inability to activate caspase-8 in response to TLR3 results in deregulated cell death. (A) Kaplan–Meyer survival curve and body temperature (mean +SD) of control (Casp8^fl^, n=5) and Casp8^ΔIEC^mice (n=10) after poly(I:C) injection. Experiments were performed four times with similar results. (B) Upper panel: Pictures exemplarily demonstrate the shortening of the intestine from a Casp8^ΔIEC^mouse and a normal intestine from a control mouse 6 h after poly(I:C) administration. Lower panel: Quantification of shortening (small intestinal length in cm). (C–E: 5 h after poly(I:C) administration): (C) Representative histological pictures of the terminal ileum of poly(I:C)-treated control and Casp8^ΔIEC^ mice. (D) Histological score and (E) quantitative PCR for *Villin* mRNA expression in the small intestine of control (fl) and Casp8^ΔIEC^mice (ΔIEC) after poly(I:C) injection. Mean values relative to hypoxanthine guanine phosphoribosyl transferase (*HPRT)* mRNA are shown +SD, n≥3. (F) Small intestinal cross-sections stained with H&E or TUNEL from poly(I:C)-treated Casp8^ΔIEC^mice after indicated time points.

**Figure 3:**
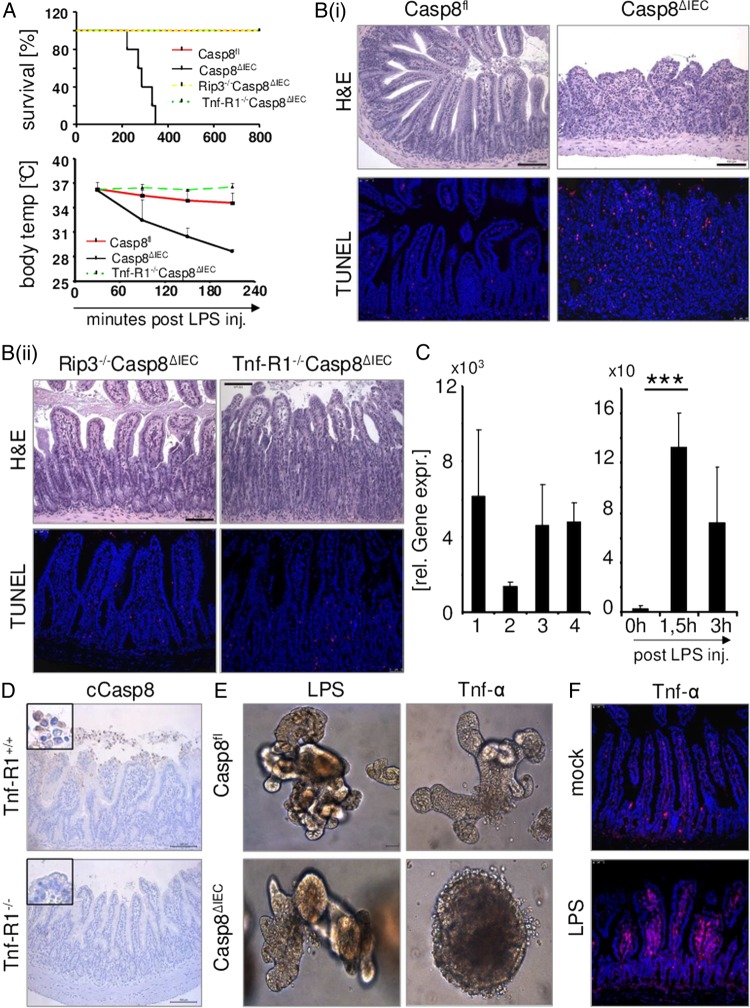
LPS-induced epithelial cell death. Control mice (Casp8^fl^, n=5), Casp8^ΔIEC^mice (n=5), Rip3^−/−^Casp8^ΔIEC^mice (n=5) and Tnf-R1^−/−^Casp8^ΔIEC^mice (n=5) were treated with LPS. Experiments were performed 3 times with similar results. (A) Kaplan–Meier survival curve and body temperature (mean +SD) of the indicated transgenic mice injected with LPS (B) i: Representative histological pictures (H&E) and ii: TUNEL staining of the small intestine of LPS treated mice (4 h after challenge). (C) LEFT: Quantitative PCR for *Villin* mRNA expression in the small intestine of control mice (lane1), Casp8^ΔIEC^mice (lane2), Rip3^−/−^Casp8^ΔIEC^mice (lane3) and Tnf-R1^−/−^Casp8^ΔIEC^mice (lane4) 4 h after LPS injection. RIGHT: Quantitative RT-PCR analysis of *Tnf*-α mRNA in the terminal ileum of control mice before and after LPS treatment (+SD, relative to *Hprt* mRNA, n≥3). (D) Cleaved caspase-8 staining of LPS-treated (90 min) Tnf-R1^+/+^mice and Tnf-R1^−/−^mice. (E) Representative images of small intestinal derived organoids from control (Casp8^fl^) and Casp8^ΔIEC^mice treated for 24 h with LPS or Tnf-α. (F) Immunofluorescence staining for Tnf-α in small intestinal sections of untreated (mock) or LPS-treated (90 min) control mice.

In order to assess a functional role of caspase-8 activation in TLR ligand-induced epithelial cell loss, we next compared control and Casp8^ΔIEC^ mice after stimulation of either TLR3 or TLR4. Whereas all control animals survived treatment with poly(I:C) or LPS, high mortality was observed in Casp8^ΔIEC^ mice already during the first hours following TLR ligand exposure (figures 2A and 3A). Similarly, TLR ligand exposure induced a rapid decrease in body temperature and body weight in Casp8^ΔIEC^ mice, whereas no such temperature or body weight decline was observed in wild-type (Casp8^fl^) animals (figures 2A and 3A, see online supplementary figure S2A). A reduced length of the small intestine was also observed in Casp8^ΔIEC^ but not in wild-type animals (figure 2B). Moreover, histological analysis demonstrated extensive villous atrophy, severe destruction of the small bowel and an excessive amount of dying epithelial cells, leading to the total destruction of the normal crypt-villus architecture in TLR3 or TLR4 triggered Casp8^ΔIEC^ mice (figures 2C,D and 3B). Loss of epithelial cells was further investigated by measuring mRNA levels of the epithelial marker villin (figures 2E and 3C). To better understand the kinetics of this highly dynamic phenomenon, we investigated gut morphology at different time points following poly(I:C) treatment. A massive amount of TUNEL-positive epithelial cells were detected already 60 min after poly(I:C) injection into Casp8^ΔIEC^ mice and peaked at 120 min (figure 2F). At 3 h, the amount of positive cells was strongly reduced accompanied by an almost total loss of the villus and crypt epithelium (figure 2F). Epithelial erosions were also observed in colon tissue of Casp8^ΔIEC^ mice, although at a lesser extent as in the small intestine (figure 5C). In summary, these data illustrate the critical role of caspase-8 in the maintenance of epithelial barrier integrity following challenge with microbial toxins.

### TLR3 and TLR4 stimulation induce excessive cell death in caspase-8-deficient epithelial cells

**Figure 4:**
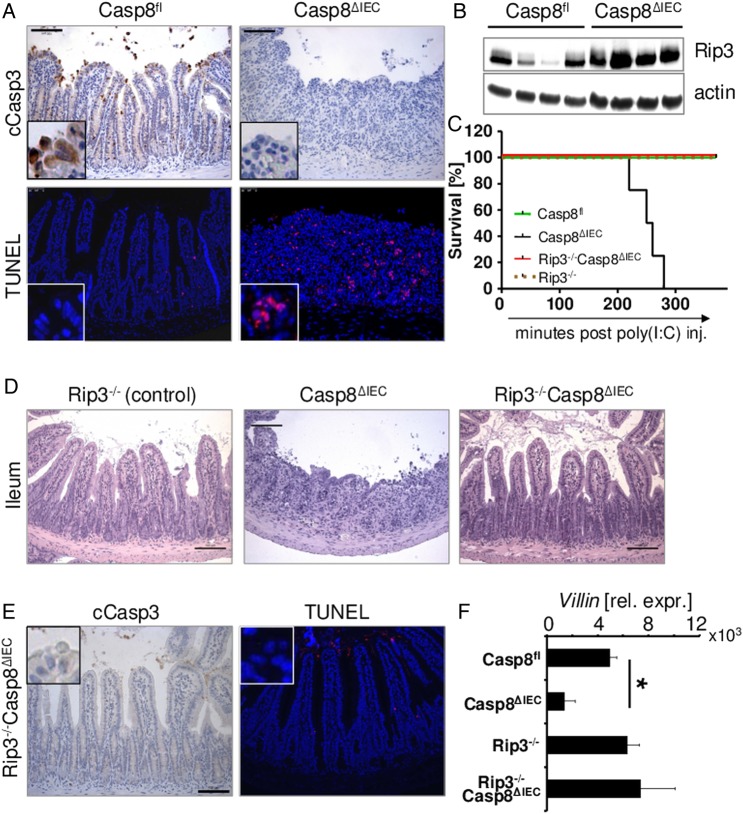
Toll-like receptor 3-induced cell death in caspase-8 deficient cells is Rip3 mediated and resembles necroptosis. (A,B and D–F: 4 h after poly(I:C) injection) (A) Representative pictures of small intestinal cross-sections from poly(I:C)-treated control (Casp8^fl^) and Casp8^ΔIEC^mice stained for TUNEL (red) and activated caspase-3 (brown). Nuclei are stained in blue. (B) Western blot for Rip3 of intestinal epithelial cell (IEC) lysates isolated from the small intestine of poly(I:C) treated control (Casp8^fl^) and Casp8^ΔIEC^mice. Actin is shown as a loading control. (C) Kaplan–Meier survival curve of control mice (Casp8^fl^, n=3), Rip3^−/−^ (n=4), Casp8^ΔIEC^mice (n=4) and Rip3^−/−^Casp8^ΔIEC^mice (n=5) after intraperitoneal injection of poly(I:C). Experiments were performed three times with similar results. (D) H&E staining of small intestine obtained from Rip3^−/−^, Casp8^ΔIEC^ and Rip3^−/−^Casp8^ΔIEC^ animals treated with poly(I:C). (E) Small intestinal cross sections of Rip3^−/−^Casp8^ΔIEC^ animals stained for TUNEL and cleaved caspase-3. (F) Quantification of IECs by measuring *Villin* expression in the ileum of control mice (Casp8^fl^), Casp8^ΔIEC^mice, Rip3^−/−^mice and Rip3^−/−^Casp8^ΔIEC^ animals after poly(I:C) treatment. (+SD, relative to *Hprt* mRNA, n≥3).

Necroptosis occurs in Paneth cells in Casp8^ΔIEC^ mice and is defined as a caspase-3 negative, TUNEL positive, Rip3-positive cell death. Accordingly, we investigated whether this form of cell death occurs in the villus epithelium of Casp8^ΔIEC^ mice following TLR stimulation. Caspase-3 activity in the small intestine of TLR ligand-treated wild-type and Casp8^ΔIEC^ mice was evaluated. By contrast with wild-type mice, which exhibited a high activity of caspase-8 and caspase-3 in exfoliated cells at the villous tip, no caspase-3-positive epithelial cells were detected in Casp8^ΔIEC^ mice (figure 4A, see online supplementary figure S1A). While only a few TUNEL-positive cells were observed in TLR ligand-treated wild-type mice, IECs of Casp8^ΔIEC^ mice showed a marked TUNEL positivity (figures 3B and 4A). Additionally, immunoblot analysis demonstrated upregulation of the necroptosis mediator Rip3 in the small intestine of poly(I:C) treated Casp8^ΔIEC^ mice, when compared to control littermates (figure 4B).

To investigate whether TLR ligands induce Rip-mediated necroptosis in caspase-8-deficient cells, we injected poly(I:C) or LPS into Rip3^−/−^Casp8^ΔIEC^ double deficient mice. By contrast with Casp8^ΔIEC^ mice, TLR ligand-challenged Rip3^−/−^Casp8^ΔIEC^ mice were protected from severe hypothermia and death, and failed to show signs of wasting disease (figures 3A and 4C, see online supplementary figure S2A). To further determine the relative contribution of Rip3-mediated necrotic cell death to gut damage, we carefully compared the bowel pathology of control and Rip3^−/−^Casp8^ΔIEC^ mice. No difference in the histology of the gut or the number of dying cells was found between both groups (figures 3B, C and 4D–F, see online supplemenatry figure S2B). Furthermore, no TUNEL- or cleaved caspase-3-positive epithelial cells were noted in Rip3^−/−^Casp8^ΔIEC^ mice (figures 3B and 4E). These results indicate that TLR3 and TLR4 ligand-induced epithelial cell death, in the absence of caspase-8, is mediated via Rip3 and that blockade of necroptosis substantially preserves epithelial integrity and prevents mortality. Collectively, our data indicate that caspase-8 is essential for controlling tissue damage during TLR-induced inflammatory cell shedding, by inhibiting necroptosis.

### Differential dependency of TLR-induced necroptosis upon Tnf-R1 signalling in vivo

**Figure 5:**
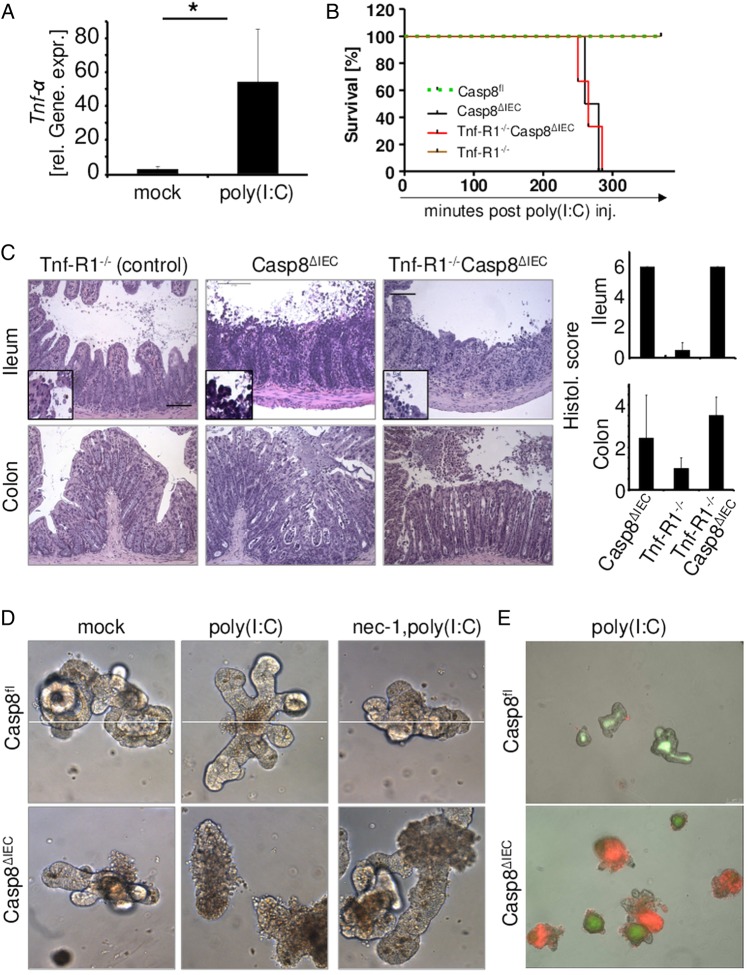
Toll-like receptor 3-induced cell death in caspase-8-deficient cells is independent of Tnf-α. (A) Quantitative expression levels of *Tnf-α* mRNA in the small intestine of mock-treated or poly(I:C)-treated wild-type animals. (+SD, relative to *Hprt* mRNA, n≥3, 2 h after poly(I:C) injection). (B) Kaplan–Meier survival curve of control mice (Casp8^fl^, n=4), Tnf-R1^−/−^(n=4), Casp8^ΔIEC^ mice (n=2) and Tnf-R1^−/−^Casp8^ΔIEC^mice (n=3) after poly(I:C) injection. Experiments were repeated three times with similar results (C) H&E staining of ileal and colon tissue sections and scoring (+SD) of intestinal tissue damage in Tnf-R1^−/−^mice, Casp8^ΔIEC^mice and Tnf-R1^−/−^Casp8^ΔIEC^mice 4 h after poly(I:C) administration. (D) Representative pictures and (E) Propidium Iodide (PI) staining of organoids derived from Casp8^fl^ and Casp8^ΔIEC^ crypts after administration of mock or poly(I:C) in the culture medium supplemented with or without nec-1 (12 h after administration).

TLR stimulation leads to the secretion of proinflammatory cytokines, such as Tnf-α. We previously reported that Tnf-α via Tnf-R1 represents a potent mediator of epithelial necroptosis in Casp8^ΔIEC^ mice, particularly in Paneth cells at the crypt base. Therefore, we evaluated the role of Tnf-α in TLR-induced epithelial cell death. Indeed, we detected significant levels of *Tnf-α* mRNA in the gut of ligand-exposed wild-type mice (figures 3C and 5A). However, parenteral LPS administration to Tnf-R1^−/−^Casp8^ΔIEC^ double-deficient mice, failed to induce signs of septic shock, including loss of body temperature and weight (figure 3A, see online supplementary figure S2A). Moreover, all Tnf-R1^−/−^Casp8^ΔIEC^ mice survived the treatment, and histological visualisation revealed an intact gut epithelium (figure 3A,B). An enhanced LPS concentration was administered to exclude a dose-dependent defect. Even after high-dose LPS challenge (mice were challenged two times with LPS), no signs of systemic inflammatory response syndrome (SIRS) were observed. Additionally, histological analysis of the gut did not detect differences in the number of shed cells between Tnf-R1^−/−^Casp8^ΔIEC^ mice and control animals (figure 3B, C, see online supplementary figure S2B).

To also assess the role of Tnf-α in LPS-induced caspase-activation, we injected LPS into Tnf-R1^−/−^ mice. No activation of caspase-8 was noted in the epithelium of LPS-challenged Tnf-R1^−/−^ mice, demonstrating that bacterial challenge induces inflammatory shedding via Tnf-R1-mediated caspase activation (figure 3D). Moreover, these data indicate that LPS-induced necroptosis in Casp8^ΔIEC^ mice is also indirectly mediated via the secretion of Tnf-α. To further determine the cellular source of Tnf-α, we used in vitro cultures of crypt organoids. LPS at concentrations up to 1000 ng/mL failed to induce epithelial cell shedding or death of organoids independent of the activation status of caspase-8 (figure 3E, see online supplementary figure 2C). However, stimulation of epithelial Tnf-R1 by addition of Tnf-α to the culture medium resulted in rapid death of caspase-8-deficient organoids but not wild-type epithelial cells (figure 3E, see online supplementary figure S2C). Notably, immunostaining of ileal tissue sections for Tnf-α revealed high expression of Tnf-α after 90 min of LPS administration in lamina propria cells adjacent to the intestinal epithelium but not in IECs (figure 3F). Thus, Tnf-α produced by non-epithelial cells upon LPS stimulation represents the necroptosis triggering factor rather than direct TLR4 signalling in IEC.

In sharp contrast with the Tnf-α-dependent effect of LPS, combined deletion of Tnf-R1 in Casp8^ΔIEC^ mice did not protect these animals from poly(I:C)-induced lethality. Tnf-R1^−/−^Casp8^ΔIEC^ mice died with a similar kinetic as compared to Casp8^ΔIEC^ mice (figure 5B). They also exhibited severe hypothermia and an even more pronounced loss of body weight. Histological analysis revealed poly(I:C)-induced destruction of the normal crypt-villus architecture, with epithelial erosion in the small intestine and the colon irrespective of the presence or absence of the Tnf-R1, suggesting that TLR3-induced gut damage occurs independent of Tnf-α (figure 5C). Besides *Tnf-α*, *type III IFN* expression is induced after TLR3 stimulation or viral infection.^20–22^ Notably, *IFN-λ* expression was increased in the small intestine of wild-type mice after poly(I:C) administration (see online supplementary figure S3B). However, IL-28Rα^−/−^ animals showed massive epithelial cell shedding associated with caspase activation indistinguishable from IL-28R-sufficient animals 2 h after poly(I:C) administration (see online supplementary figure S3B).

To exclude the contribution of other mediators produced by gut immune cells, intestinal organoids from control (Casp8^fl^) and Casp8^ΔIEC^ mice were treated in vitro with poly(I:C). By contrast with the observation made after LPS stimulation, targeting of TLR3 by administration of poly(I:C) to the organoid culture, resulted in rapid epithelial cell death of caspase-8 deficient organoids but not of organoids derived from control mice (figures 5D, E see online supplementary figure S3A). Moreover, pretreatment of caspase-8-deficient organoid cultures with the Rip1-specific inhibitor necrostatin-1 (nec-1), an inhibitor of necroptosis, readily inhibited poly(I:C)-induced epithelial necrosis (figure 5F, see online supplementary figure S3A). Thus, our data suggest that poly(I:C)-induced cell death in caspase-8-deficient epithelial cells is independent of Tnf-R1 or IL-28R signalling but is rather directly mediated via the TLR3-Rip kinase1/3 pathway.

It has previously been shown that TLR3 and TLR4 activation triggers the recruitment of TRIF, which interacts with Rip1 and caspase-8 via the RHIM domain of Rip1. Consistent with published results,^14^ we found that TRIF^−/−^ mice were protected from poly(I:C)-induced epithelial gut damage (see online supplementary figure S4A). No epithelial cell shedding or signs of SIRS were observed in poly(I:C)-treated TRIF^−/−^ animals. Moreover, poly(I:C) did not induce the disturbance of E-cadherin expression in adherence junctions or the activation of caspase-8 and caspase-3 (see online supplementary figure S4A–C) in TRIF-deficient epithelial cells. To determine if poly(I:C)-induced necroptosis was also mediated via TRIF, we generated small intestinal organoids derived from TRIF-deficient mice. Epithelial cell organoids were treated in vitro with poly(I:C) in the presence or absence of the caspase inhibitor zVAD-fmk. This allowed us to directly investigate the response of epithelial cells lacking caspase activity and/or TRIF-dependent signalling in response to poly(I:C). As shown by light microscopy and also PI staining, addition of poly(I:C) in combination with zVAD-fmk to the culture medium resulted in rapid cell death of wild-type but not TRIF-deficient organoids (see online supplementary figure S4D, E).

### Caspase-8 activity is important in the maintenance of epithelial barrier function after TLR challenge

**Figure 6:**
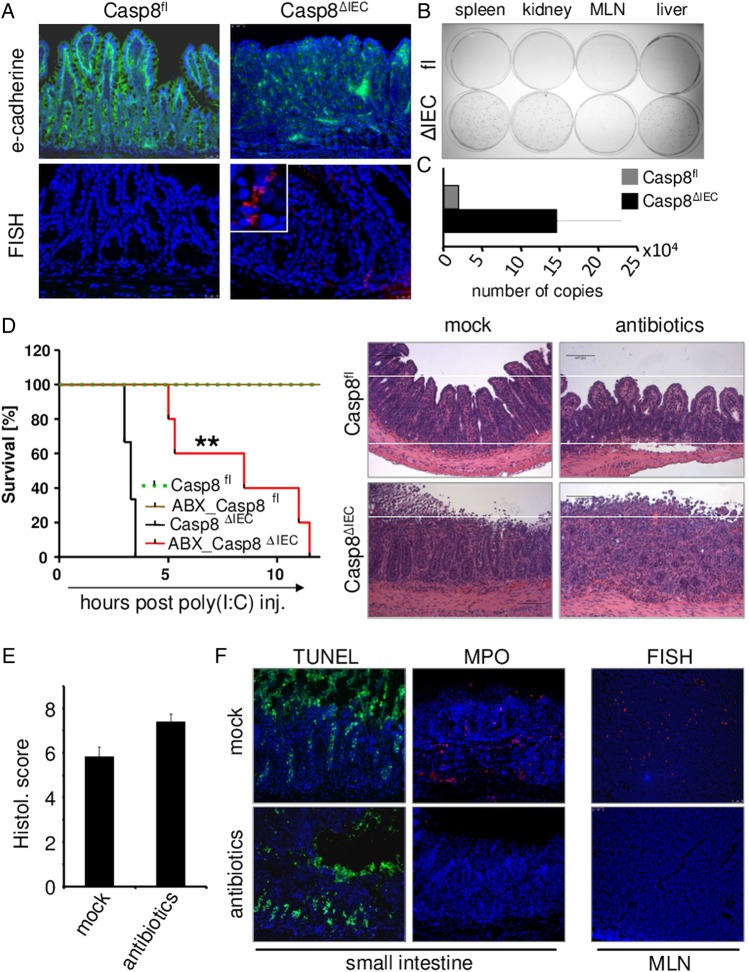
Caspase-8 activity is important in maintaining the epithelial barrier. (A–C, E and F: 4 h after poly(I:C) injection) (A) Immunostaining of tissue sections of the terminal ileum of poly(I:C)-treated control (Casp8^fl^) and Casp8^ΔIEC^mice for E-cadherin (green), upper panel. Fluorescence in situ hybridization (FISH) staining for eubacterial RNA in tissue sections of the terminal ileum of poly(I:C)-treated control and Casp8^ΔIEC^mice, lower panel. (B) Bacterial culture after plating homogenised tissues (MLN, mesenteric lymph nodes) of control (fl) and Casp8^ΔIEC^mice (ΔIEC) treated with poly(I:C) i.p. (C) Quantification of bacterial burden in the liver of poly(I:C) treated animals. (D) Kaplan–Meier survival curve and H&E staining of small intestinal tissue sections of mice left untreated (Casp8^fl^, n=3; Casp8^ΔIEC^, n=3) or treated with antibiotics (Casp8^fl^, n=4; Casp8^ΔIEC^ mice, n=5) prior to intraperitoneal injection of poly(I:C). Experiments were performed three times with similar results. (E) Histological score of the small intestine of Casp8^ΔIEC^mice treated with or without antibiotics prior to poly(I:C) injection. (F) Representative images for TUNEL, MPO in tissue sections of the terminal ileum and FISH staining in mesenteric nodes of untreated or antibiotic-treated Casp8^ΔIEC^ mice injected with poly(I:C).

Given that poly(I:C)-induced mortality of Casp8^ΔIEC^ mice within only a few hours, we examined whether this was a direct effect of TLR3-signalling or if bacteria are involved, since we could identify that intestinal damage in these mice was accompanied by a breakdown of the intestinal barrier, as demonstrated by staining for the adherence junction protein E-cadherin (figure 6A).

In line with defective intestinal barrier function in poly(I:C)-challenged Casp8^ΔIEC^ mice, fluorescence in situ hybridisation (FISH) with an eubacterial rRNA-specific probe revealed enhanced translocation of intestinal bacteria from the luminal side into subepithelial areas of the lamina propria (figure 6A). Even more striking, Casp8^ΔIEC^ mice showed a systemic spread of luminal bacteria into different organs suggesting that barrier defects in caspase-8-deficient mice might be responsible for the lethal outcome (figure 6B, C). To confirm that bacterial spread was responsible for the high mortality observed in Casp8^ΔIEC^ mice, we severely reduced the intestinal microbiota by treating Casp8^ΔIEC^ mice with a combination of different antibiotics prior to administration of the stimulus poly(I:C). As expected, antibiotic treatment significantly prolonged the survival of Casp8^ΔIEC^ mice after poly(I:C) treatment (figure 6D). Additionally, antibiotic treatment reduced numbers of infiltrating polymorphonuclear cells and the number of bacteria in mesenteric lymph nodes, while at the same time, poly(I:C)-induced gut damage was comparable between both groups (figure 6D–F). Collectively, these results suggest that caspase-8 maintains epithelial barrier integrity following poly(I:C) challenge, and thus prevents a toxic spread of luminal bacteria to systemic body sites.

## Discussion

Defects in intestinal barrier function due to tight junction loss or cell death-driven cell shedding and barrier loss can exacerbate or directly trigger intestinal inflammation.^1^ ^23^ While previous studies have focussed on the molecular regulation of apoptosis and necroptosis in the intestinal epithelium, triggering factors for caspase-8-dependent and caspase-8-independent cell death in the small intestine are not well defined. The only strong inducer of small intestinal necroptosis that has been reported so far is Tnf-α. However, additional deletion of Tnf-R1 in Casp8^ΔIEC^ mice could not inhibit Paneth cell necroptosis and inflammation, suggesting that cell death in these animals is either activated by Tnf-R1 independent pathways or that cell death in vivo is triggered by redundant death-inducing signals.^15^ In line with a complex role of Tnf in the regulation of epithelial cell death, in a different model using DSS-induced colitis, it has been demonstrated that the ablation of Tnf-R1 resulted in increased apoptosis of colonic epithelial cells, indicating that caspase-8-associated cell death can be triggered by Tnf-R1 independent pathways, and that under certain conditions, Tnf-R signalling might have a protective function.^24^ Our data now provide in vivo evidence for redundant death-inducing signals, and highlight TLR ligands as potent activators of caspase-8-dependent and caspase-8-independent epithelial cell death.

Our results clearly demonstrate that activation of caspase-8 by ligand binding to TLRs is associated with increased epithelial cell loss in wild-type animals. Challenge of mice lacking caspase-8 selectively in the intestinal epithelium via TLR3 or TLR4 ligation did not protect them from epithelial loss but, instead, caused severe gut injury due to excessive Rip3-mediated necroptotic cell death. By contrast with caspase-8-mediated inflammatory cell shedding, epithelial necroptosis resulted in a total disruption of the mucosal surface, finally causing a lethal systemic inflammatory shock in Casp8^ΔIEC^ mice. Thus, our data for the first time show a crucial role for caspase-8-mediated Rip3 inhibition in controlling the epithelial response to TLR ligands.

Surprisingly, our study identified that TLR3 and TLR4 both trigger epithelial cell death, but that the signalling pathways differ. Caspase-8 activation and gut damage, upon TLR4 stimulation, was prevented in Tnf-R1^−/−^ mice, demonstrating that LPS-induced cell death is strictly dependent on Tnf-α. Additionally, we discovered that Tnf-R1^−/−^Casp8^ΔIEC^ mice were protected from LPS-induced cell death, demonstrating that TLR4-mediated necroptosis is also mediated via this cytokine. The fact that Tnf-α is produced upon LPS administration by gut immune cells and not by IECs, indicates that bacterial toxins do not directly induce cell death via TLR4 on epithelial cells. This is supported by only low expression of TLR4 in IECs and the fact that organoids did not show cell death in response to LPS. Therefore, initial recognition of LPS associated with caspase-8 or Rip3 activation occurs via TLR4 ligation in lamina propria cells rather than epithelial cells (see online supplementary figure S5). One may speculate that in the situation of an increased intestinal permeability, an enhanced paracellular flux of intestinal pathogens may result in the activation of innate immune cells promoting their secretion of Tnf-α which subsequently induces epithelial cell death via Tnf-R1 on epithelial cells (see online supplementary figure S5). Given that the epithelium is constantly exposed to bacteria-derived TLR ligands, it seems that it would be perilous if the commensal flora could directly trigger caspase-8-dependent cell loss via TLR4 on epithelial cells. Our findings in IECs are in contrast with a recent publication in which it was shown that LPS is able to activate necroptosis in FADD-deficient dendritic cells directly via the TLR4 pathway.^25^ The fact that different cell types use different signalling pathways for programmed necrosis suggests adapted strategies in the defence against pathogens.

The observation that Tnf-R1 deficiency did not protect mice from TLR3-mediated cell death indicates that viral products, such as dsRNA, can directly mediate caspase-8-dependent cell death. These results are in line with a previous publication demonstrating that dsRNA rapidly induces epithelial shedding associated with caspase-8 activation independent of Tnf-α production after TLR3 ligation.^14^ Our experimental data confirm that TLR3 can directly signal into IECs independent upon Tnf. Our data provide an extensive characterisation of caspase-8-dependent and caspase-8-independent cell death in response to TLR3 ligation and, for the first time, indicate that TLR3 signalling can directly induce Rip-dependent necroptosis in vivo.

Previous in vitro studies indicated that recognition of poly(I:C) by TLR3 under certain conditions can result in the formation of an intracellular platform containing the TRIF adapter that directly recruits Rip1 and caspase-8 to the TLR3, suggesting that this complex is able to mediate caspase-dependent and caspase-independent cell death pathways.^26^ In line with this, we found that TRIF was required for poly(I:C) induced intestinal damage, as reported before by others.^14^ We could now extend these previous findings by discovering that TRIF-deficiency protected the intestinal epithelium from Rip-mediated necroptosis. Thus, our data provide evidence that dsRNA-induced apoptosis and necroptosis are strictly dependent on this adapter protein. Additionally, we discovered that poly(I:C)-induced necroptosis proceeds via assembly of the Rip1–Rip3 complex, demonstrated by the fact that deletion of Rip3 in vivo (Rip3^−/−^Casp8^ΔIEC^ double deficient mice) and inhibition of Rip1 in vitro (nec-1) were sufficient to block cell death. Taken together, our data for the first time demonstrate in vivo that viral products, such as dsRNA induce intestinal epithelial necroptosis independent of immune cells, in a situation in which caspase-8 activity is impaired by directly targeting TLR3 on IECs (see online supplementary figure S5).

In summary, our data highlight a crucial role for caspase-8-mediated Rip3 inhibition in controlling the epithelial response to bacteria and virus-associated molecular patterns. This has important clinical implications since therapies that inhibit apoptosis are likely to carry substantial toxicity in the intestinal epithelium, and might induce necroptosis and inflammation. Future therapeutic strategies might benefit from the simultaneous inhibition of apoptosis and necroptosis.

## Supplementary Material

Web supplement

Web figures
