# Supplementary material for: Caspase-8 controls the gut response to microbial challenges by Tnf-α-dependent and independent pathways
Source: Gut. 2014 Jun 24;64(4):601–10. doi: 10.1136/gutjnl-2014-307226 (PMC4392221; doi:10.1136/gutjnl-2014-307226)
Supplement: Web supplement [file gutjnl-2014-307226-s1.pdf]

Supplementary Information:

Suppl. Figure 1: Caspase-mediated inflammatory shedding in response to viral triggers

(a) Small intestinal cross sections of wild-type mice after i.p. injection of poly(I:C) or LPS stained with H&E or for cleaved caspase-8 and cleaved caspase-3. (b+c) Quantitative RT-PCR for *Villin* mRNA expression in different bowel segments (SI=small intestine, Co=colon) 180 minutes after poly(I:C) (b) or 90 minutes after LPS (c) administration. Mean values relative to *HPRT* mRNA are shown +SD, n≥3.

Suppl. Figure 2: LPS mediated cell death necroptosis is independent of Tnf-α.

(a) Relative weight of indicated mice 4,5 hours after LPS administration. Control mice (Casp8^{fl}, n=5), Casp8$^{\Delta IEC}$mice (n=5), Rip3$^{-/-}$ (n=4), Rip3$^{-/-}$Casp8$^{\Delta IEC}$mice (n=5) and Tnf-R1$^{-/-}$ Casp8$^{\Delta IEC}$mice (n=5), upper panel. Kaplan-Meier survival curve of Rip3$^{-/-}$ (n=4) and Tnf-R1$^{-/-}$ (n=2) injected with LPS, lower panel. (b) Representative pictures of small intestinal cross sections from LPS-treated Rip3$^{-/-}$ and Tnf-R1$^{-/-}$ mice stained for TUNEL (red). Nuclei are stained in blue. (4 hours after challenge). (c) Representative pictures and PI staining (red) of organoids derived from Casp8$^{\Delta IEC}$ and Casp8^{fl} crypts after administration of LPS or Tnf-α to the culture medium (LPS, 24 hours after administration, Tnf-α; 8 hours after administration).

Suppl. Figure 3: TLR3-induced cell death in caspase-8 deficient cells is independent of type III interferons.

(a) Representative pictures and PI staining (red) of organoids derived from Casp8$^{\Delta IEC}$ and Casp8^{fl} crypts after administration of poly(I:C) in the culture medium supplemented with or without nec-1. (24 hours after administration) (b) GRAPH: Quantitative expression levels of *IFN-λ* mRNA in the small intestine of wild-type animals before, 2 and 4 hours after poly(I:C) administration. Mean values relative to *HPRT* are shown, +SD, n=3. PICTURES: Small intestinal cross sections of IL-28Rα$^{-/-}$mice after i.p. injection of poly(I:C) (2 hours) stained with H&E or for cleaved caspase-8 and cleaved caspase-3.

Suppl. Figure 4: TLR3-mediated caspase-8 activation is induced via the TLR adaptor molecule TRIF

(a-c: 2 hours after poly(I:C) injection) (a) Small intestinal sections obtained from mock or poly(I:C)-treated TRIF$^{-/-}$mice were stained with H&E or anti-E-cadherin antibody. (b) Staining for activated caspase-8 in the intestinal epithelium of poly(I:C)-treated control (TRIF$^{+/+}$) and TRIF$^{-/-}$mice. (c) Immunoblot analysis of caspase-8 and caspase-3 cleavage in intestinal epithelial cells isolated from TRIF$^{-/-}$animals treated with or without poly(I:C). Actin staining was added as a loading control. (d) Representative microscopic pictures of control (TRIF$^{+/+}$) and TRIF deficient organoids treated for 24 h with poly(I:C) in combination with the caspase inhibitor zVAD. Experiments were performed three times with similar results. (e) PI staining (red) of control (TRIF$^{+/+}$) and TRIF deficient organoids treated for 24 h with poly(I:C) in combination with the caspase inhibitor zVAD with or without nec-1.

Suppl. Figure 5: Model summarizing the potential mechanism by which LPS or poly(I:C) induce necroptosis in the caspase-8 deficient epithelium.

Bacterial products, like LPS are recognized by mononuclear cells expressing TLR4, resulting in the production of Tnf. Tnf ligation by Tnf-R1 on epithelial cells induces the phosphorylation of RIP kinases, which drive necroptosis. Poly(I:C) can directly activate the RIP kinases by activation the TLR3-TRIF pathway in intestinal epithelial cells, promoting necroptosis. In this case necroptosis can be triggered independent of mononuclear cells of the lamina propria.

Proteinisolation

For the isolation of intestinal epithelial cells from gut samples, the intestine was washed free of stool in PBS and carefully inverted. Inverted gut tissue was incubated in mammalian protein extraction reagent (Thermo Scientific) supplemented with protease and phosphatase inhibitor tablets (Roche) and incubated on ice for 1 hour.

Subsequently, the gut tissue was removed and isolated IEC-protein extracts were pelleted by centrifugation for 20 minutes at 14000 rpm.

Antibiotic therapy:

To deplete intestinal microflora, mice were gavaged every 12 hours with a combination of metronidazole (100mg/kg, Braun), neomycin (0,1mg/kg, Roth) and vancomycin (50mg/kg, Hikma Farmaceutica) for 4 days. Ampicillin was added to the drinking water at a concentration of 1mg/ml (Ratiopharm). Successful antibiotic therapy was verified by plating fresh faeces diluted in sterile PBS on LB agar plates.

FISH Staining:

In situ hybridization of bacterial rRNA on glass slides was performed as previously described [w1].

Supplementary references:

w1 Becker C; Wirtz S, Blessing M, Pirhonen J, Strand D, Bechthold O, *et al.* Constitutive p40 promoter activation and IL-23 production in the terminal ileum mediated by dendritic cells. J. Clin. Invest. 2003,**112**, 693–706
