# Supplementary figures and images for: Caspase-8 controls the gut response to microbial challenges by Tnf-α-dependent and independent pathways
Source: Gut. 2014 Jun 24;64(4):601–10. doi: 10.1136/gutjnl-2014-307226 (PMC4392221; doi:10.1136/gutjnl-2014-307226)

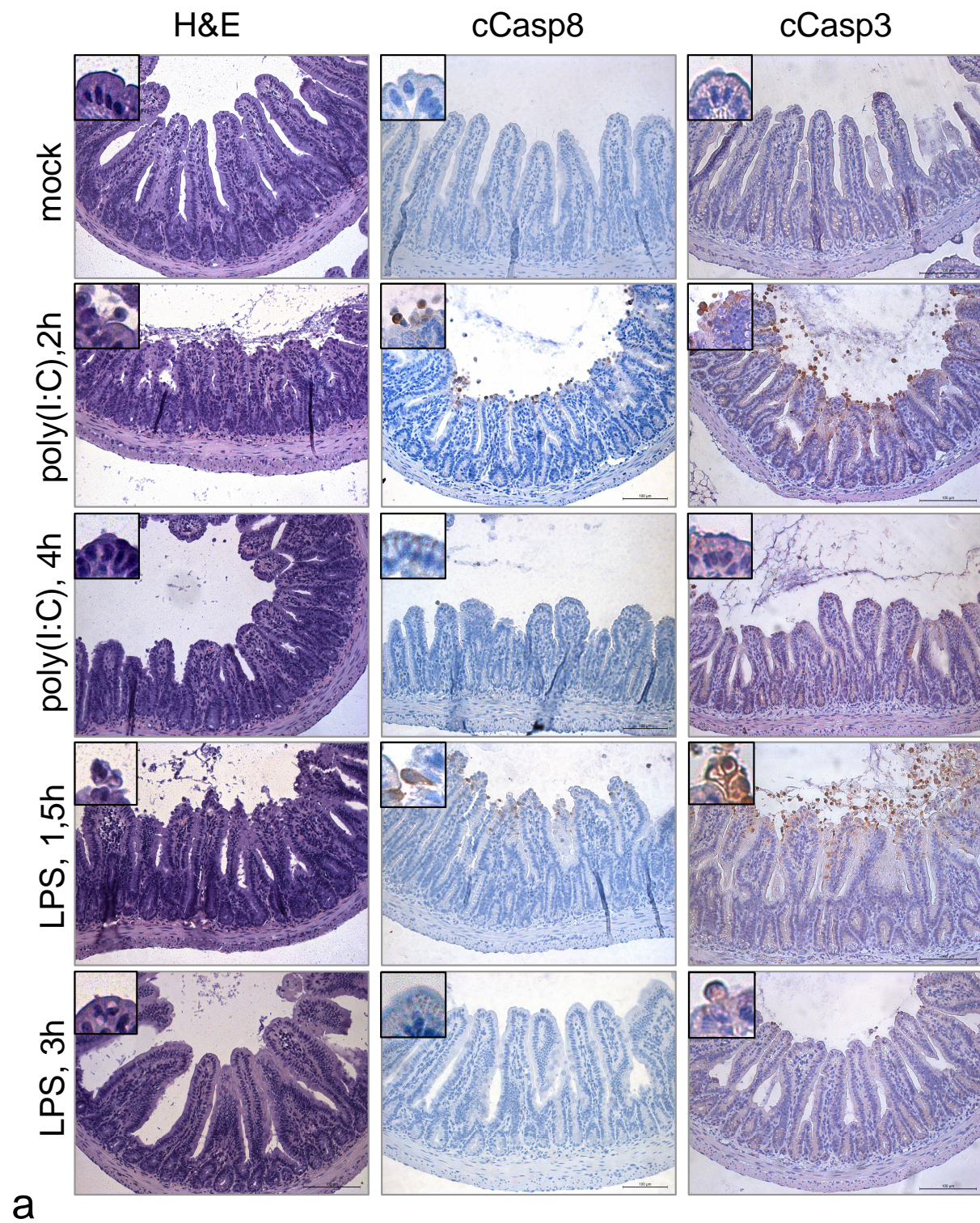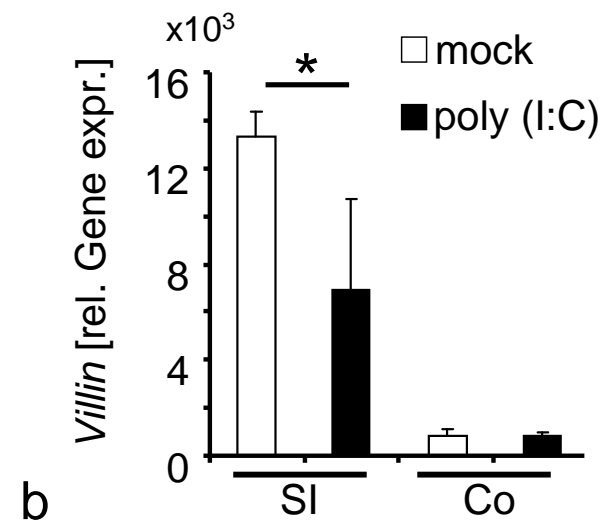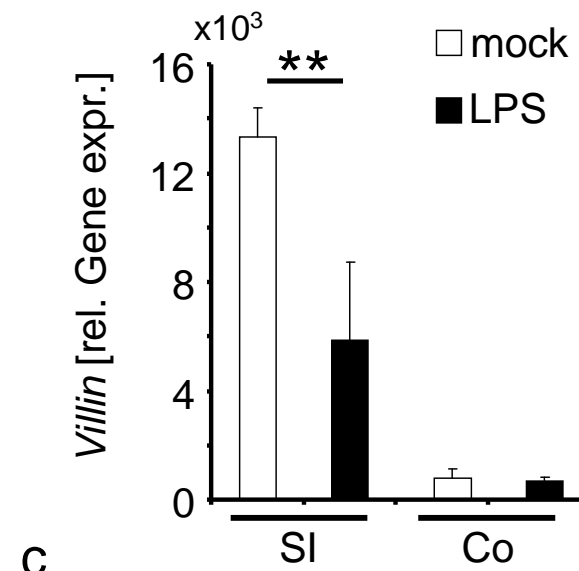

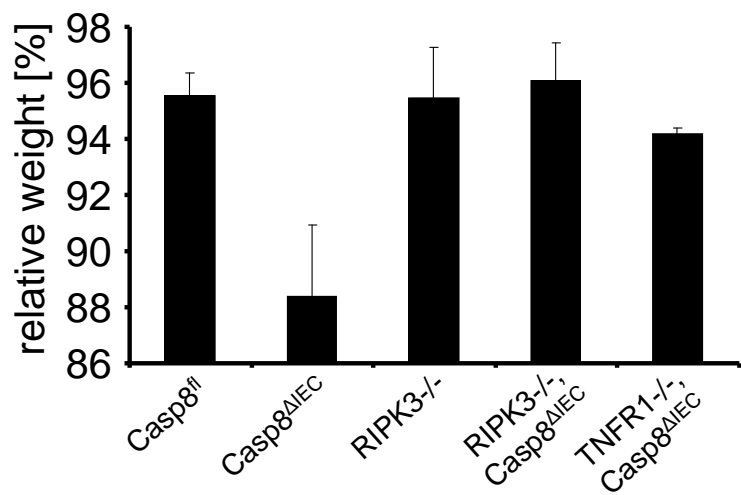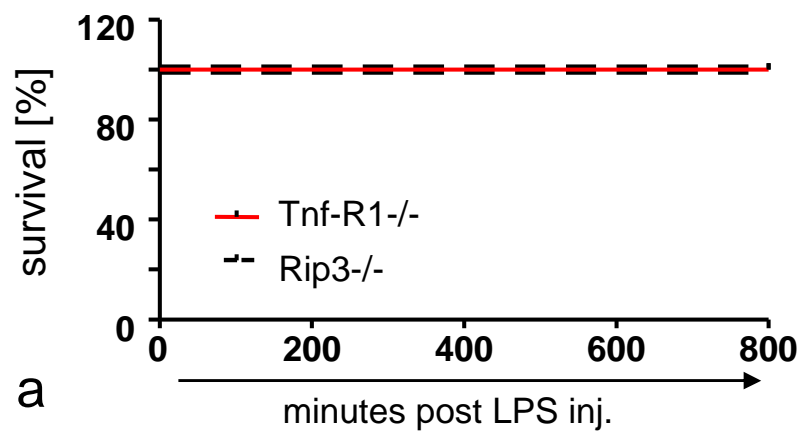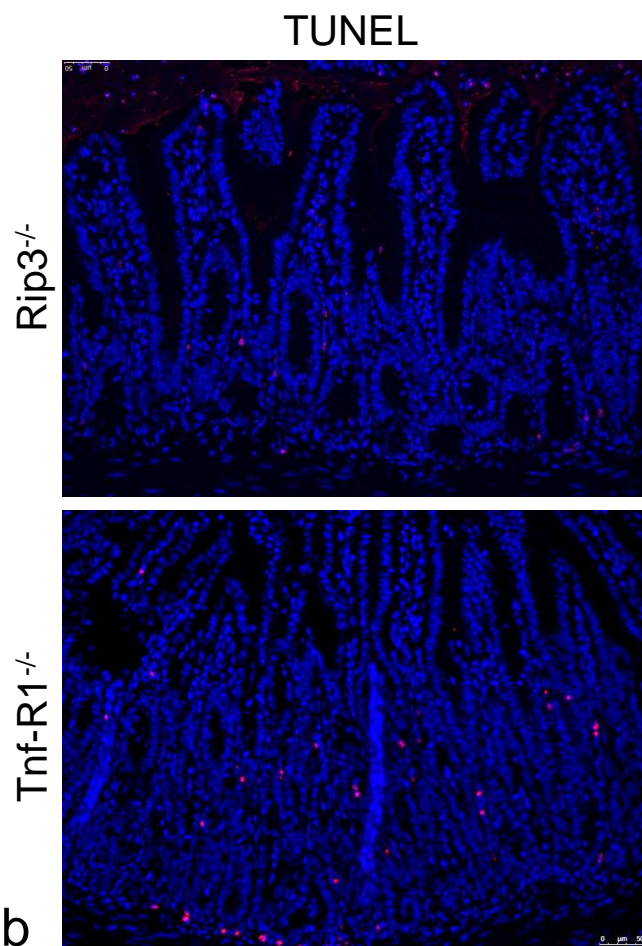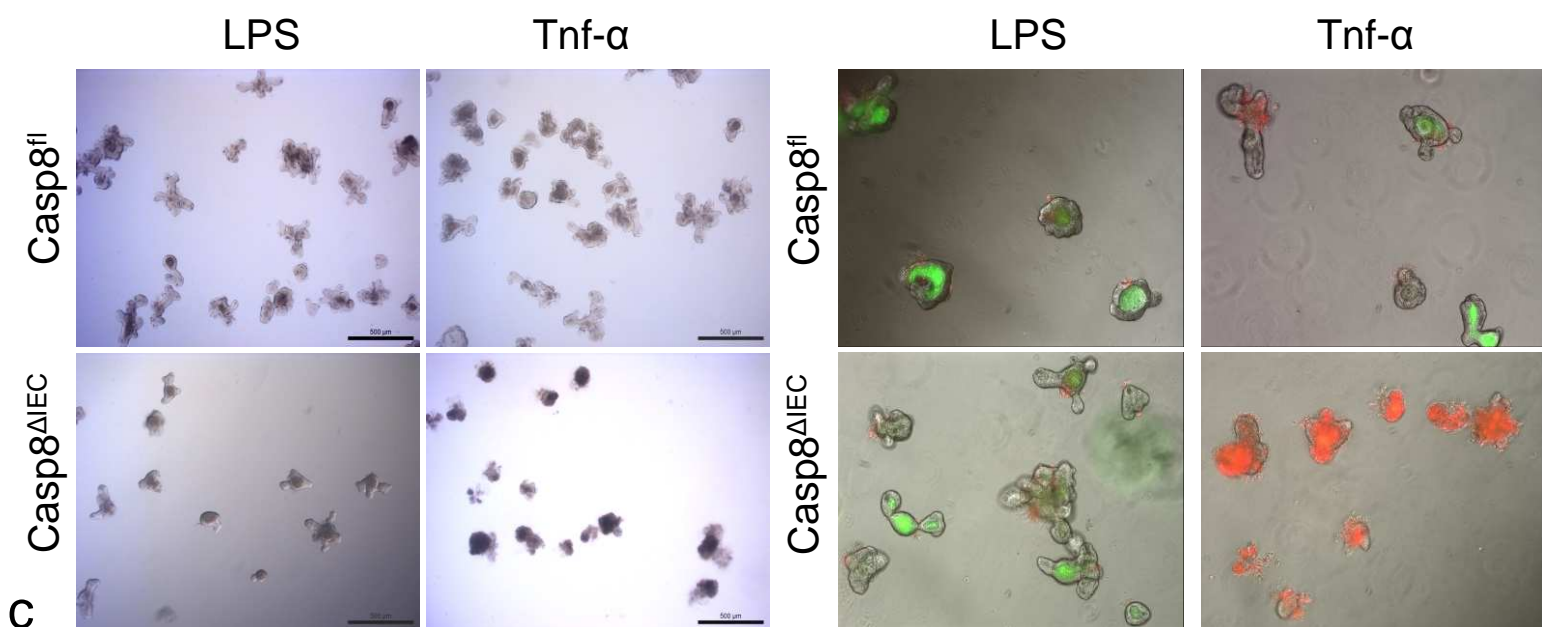

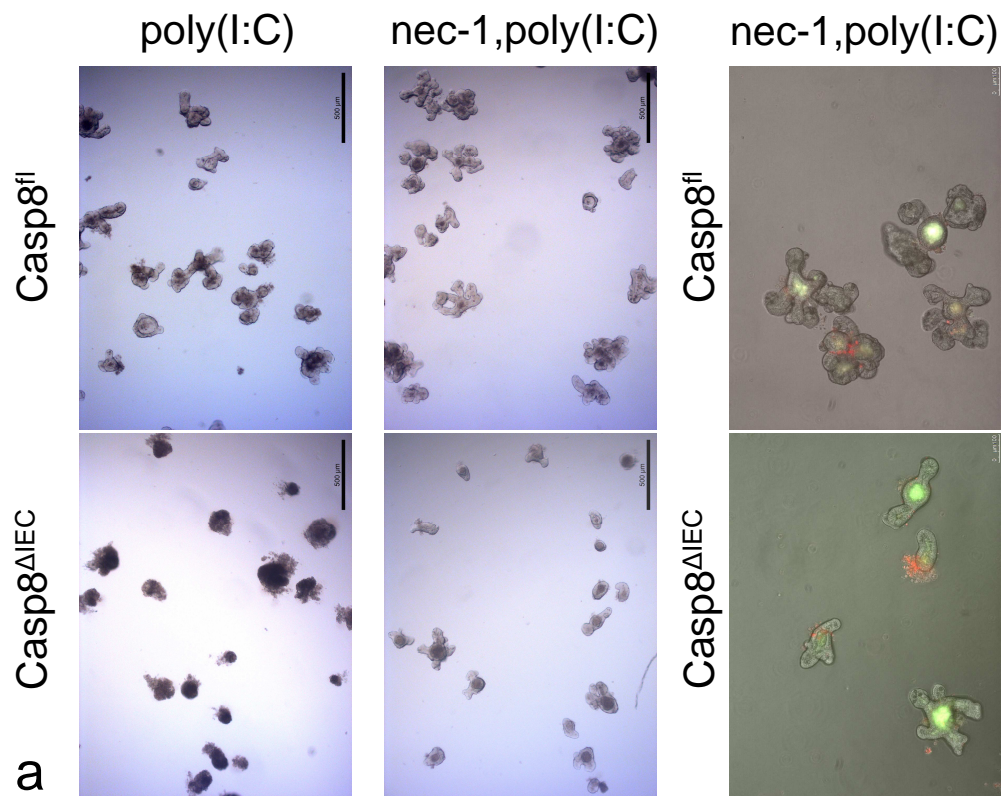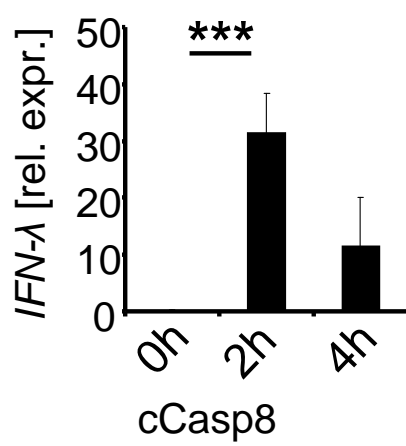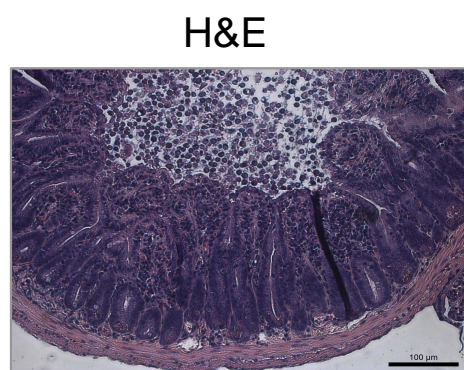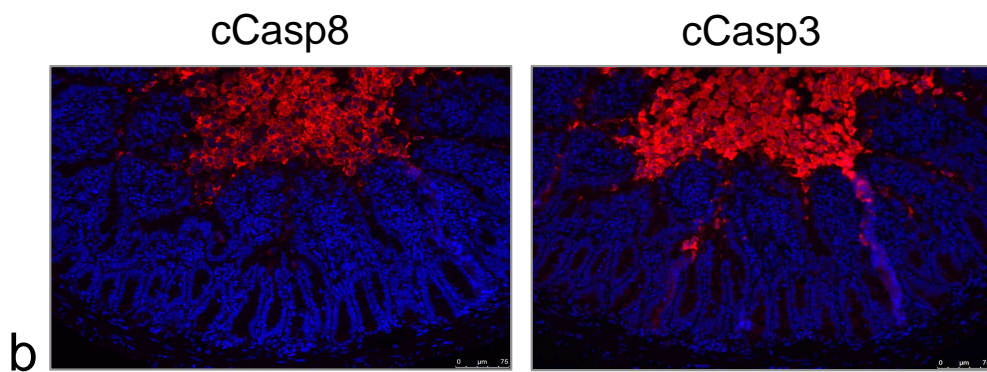

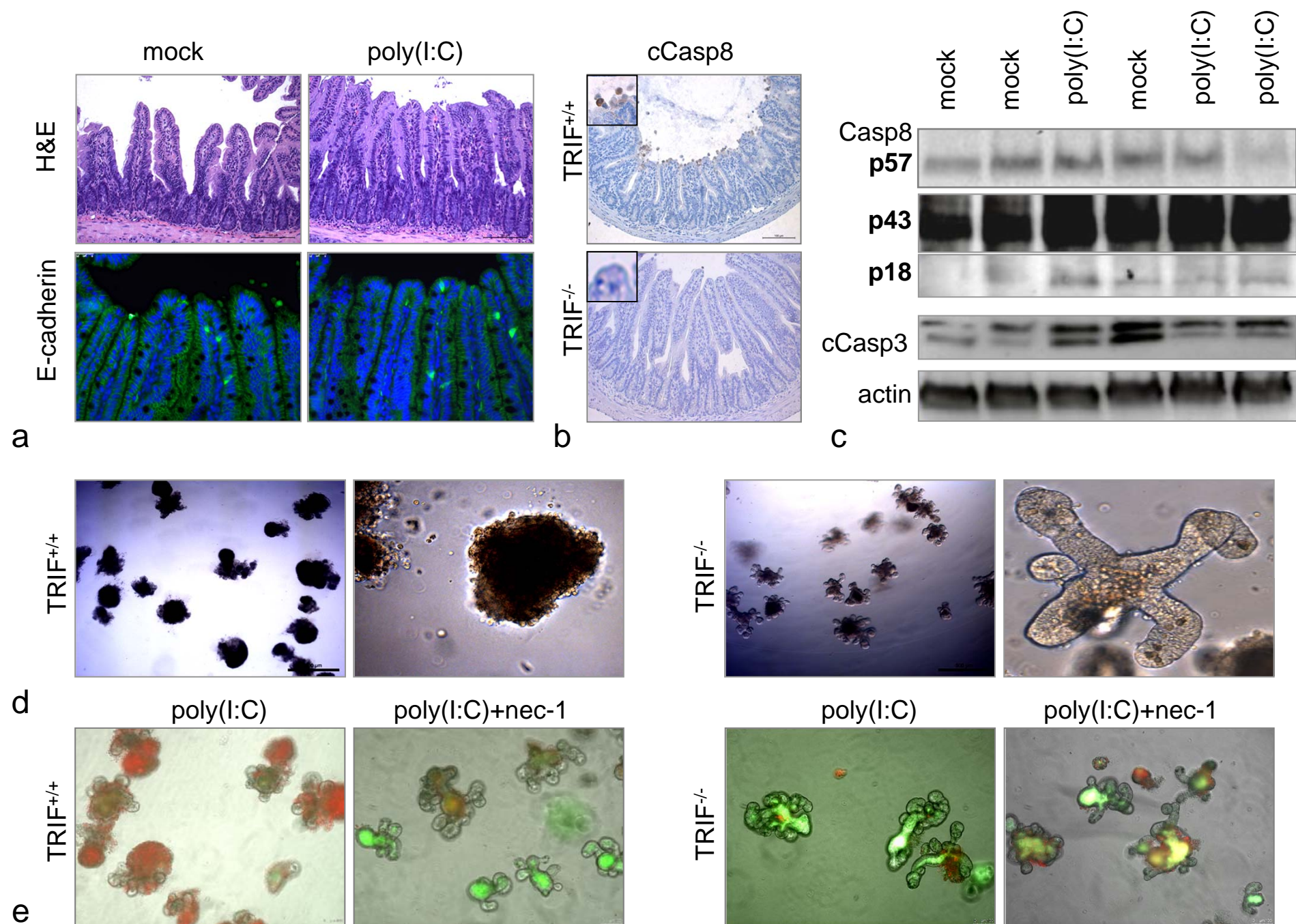

Suppl. Fig.4

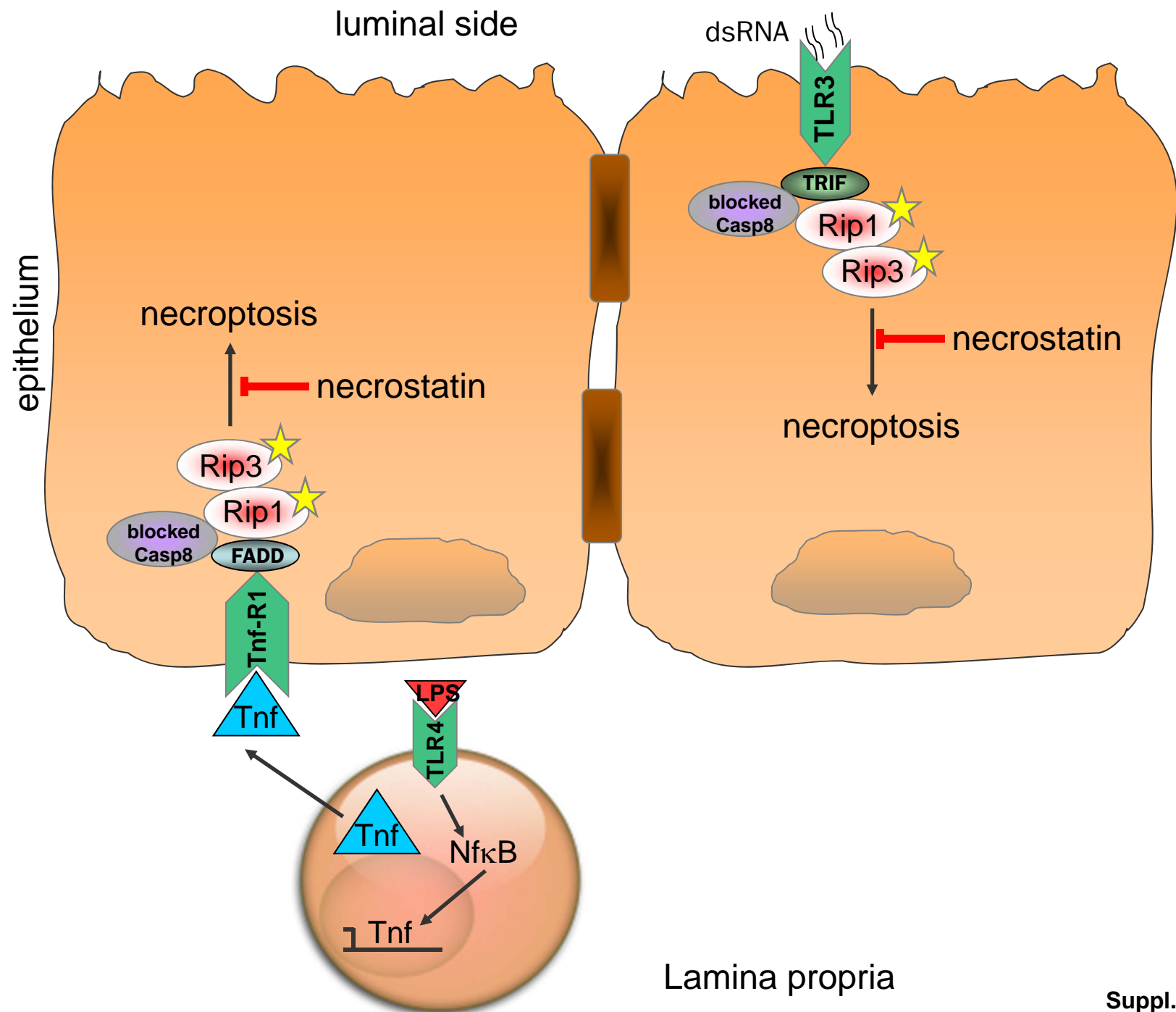

Suppl. Fig.5

Supplement: Web figures [file gutjnl-2014-307226-s2.pdf]
